# Therapeutic Strategies and Oncological Outcome of Peritoneal Metastases from Lung Cancer: A Systematic Review and Pooled Analysis

**DOI:** 10.3390/curroncol30030224

**Published:** 2023-03-01

**Authors:** Leandro Siragusa, Sara Di Carlo, Alessia Fassari, Bruno Sensi, Camilla Riccetti, Luciano Izzo, Giuseppe Cavallaro, Enrico Fiori, Paolo Sapienza, Letizia Mallia, Graziano Pernazza, Simone Sibio

**Affiliations:** 1 Department of Surgical Sciences, University of Rome “Tor Vergata”, Viale Oxford 81, 00133 Rome, Italy; 2 Department of Surgery, Sapienza University of Rome, “Umberto I” University Hospital, Viale del Policlinico 155, 00161 Rome, Italy; 3 Sapienza University of Rome—ASL Roma 6—Via borgo Garibaldi 12, Albano Laziale, 00041 Rome, Italy; 4 San Giovanni Hospital—Via dell’Amba Aradam 8, 00184 Roma, Italy

**Keywords:** lung cancer, peritoneal metastases, peritoneal carcinomatosis, palliative chemotherapy, cytoreductive surgery, HIPEC

## Abstract

The peritoneum is an unusual site of metastases from lung cancer, and optimal management at the moment remains unclear and mostly based on palliative strategies. Therefore, the aim of the study was to investigate demographic characteristics, management and overall survival of patients with peritoneal metastases from lung cancer (PCLC). A PRISMA-compliant systematic review and pooled analysis was performed searching all English studies published until December 2022. PROSPERO, CRD42022349362. Inclusion criteria were original articles including patients with peritoneal carcinomatosis from lung cancer, specifying at least one outcome of interest. Exclusion criteria were being unable to retrieve patient data from articles, and the same patient series included in different studies. Among 1746 studies imported for screening, twenty-one were included (2783 patients). Mean overall survival was between 0.5 and 5 months after peritoneal carcinomatosis diagnosis and 9 and 21 months from lung cancer diagnosis. In total, 27% of patients underwent first-line or palliative chemotherapy and 7% of them surgery. Management differs significantly among published studies. The literature on PCLC is scarce. Its incidence is low but appears to be substantially rising and is likely to be an underestimation. Prognosis is very poor and therapeutic strategies have been limited and used in a minority of patients. Subcategories of PCLC patients may have an improved prognosis and may benefit from an aggressive oncological approach, including cytoreductive surgery. Further investigation would be needed in this regard.

## 1. Introduction

Lung cancer (LC) is one of the most common malignancies in the world today. For instance, the American Cancer Society estimated that the statistics for lung cancer in the United States for 2022 were 236,740 new cases and about 130,180 deaths [1].

Metastatic disease is already present at diagnosis in about 40% of patients with the most common sites being bone, liver, brain, and adrenal gland, while gastrointestinal sites are much more uncommon [2,3]. Peritoneal carcinomatosis from lung cancer (PCLC) is indeed a quite rare occurrence, often perceived as a sign of end-life stage, also demonstrated by poor prognostic outcomes [4,5,6]. However, new molecular targets and therapies, and the increasing incidence at earlier stages due to the increased awareness and accuracy of diagnostic methods are now more dutifully raising questions on management and possible treatment [7,8,9,10].

At the moment, the literature is scarce with low-grades of evidence and few patients included, and no published guideline suggesting a specific pathway other than the standard care of lung cancer metastatic disease. Furthermore, no systematic review on the topic exists nor does a pooled analysis of PCLC patients.

Therefore, the aim of this systematic review is to investigate characteristics, management and overall survival of PCLC patients.

## 2. Materials and Methods

This is a systematic literature review performed in accordance with the current Preferred Reporting Items for Systematic Reviews and Meta-analyses (PRISMA) guidelines [11].

This review was registered in PROSPERO CRD42022349362.

### 2.1. Search Strategy

Searches were conducted for all English language full-text articles published until December 2022. The following database sources were searched: PubMed (MEDLINE), Cochrane Library, Web of Science.

The following free term combination was used: (peritoneal carcinomatosis), (peritoneal neoplasm), (gastrointestinal metastases), (lung cancer), (lung neoplasia).

Records were screened for relevance based on their title and abstract, and successively the full text of the remaining articles was analysed. Furthermore, the references list of each selected article was analysed to identify additional relevant studies.

### 2.2. Study Selection

Inclusion criteria were as follows: (1) original articles (retrospective, prospective, randomised clinical trials), case series and report; (2) articles including patients with PC from LC; (3) articles specifying at least one outcome of interest.

Exclusion criteria were as follows: (1) unable to retrieve patient data from articles; (2) meeting abstract; (3) same patient series included in different studies. In the latter case, only the most recent article was included.

### 2.3. Data Extraction and Synthesis

Two authors (A.F. and L.S.) independently screened each record from full text articles for eligibility, and extracted the data, including quality analysis. Disagreement was resolved by discussion and consensus; if no agreement was reached, a third author was consulted (S.S.).

### 2.4. Outcome Measures

Primary outcomes were patient management and overall survival from LC diagnosis and PM onset. Patient management included rate of chemotherapy and surgery and specific chemotherapy regimen utilised.

Baseline characteristics analysed were age, sex, ascites, former smoker status, PC incidence in lung cancer, time from LC diagnosis to PC, stage at diagnosis, presence and type of other metastases, tumour histology and mutations.

Additionally, a sub-analysis comparing synchronous versus metachronous PCLC patient characteristics was performed.

### 2.5. Quality Assessment

Study quality was assessed using Newcastle Ottawa Scale (NOS). NOS is an assessment tool used to measure the quality of non-randomized studies included in systematic reviews. Each article was assessed for 9 parameters, each awarding up to 1 point, with a maximum total score of 9 points [12].

### 2.6. Statistical Analysis

Categorical data are reported as absolute numbers with percentage; continuous data are reported as median with ranges. Data were pooled and descriptive statistics were produced from the dataset. A pooled analysis was performed where categorical and continuous data were reported as median, range and percentages. There was no comparative statistical analysis.

## 3. Results

### 3.1. Systematic Search

The initial database search identified 1746 articles; 1175 were duplicates, and after screening of title and abstract, 539 dealing with other subjects were excluded. After full-text reading of twenty-eight eligible articles, a further seven were excluded owing to inability to retrieve patient data. Twenty-one studies met the inclusion criteria and were finally selected for the systematic review [13,14,15,16,17,18,19,20,21,22,23,24,25,26,27,28,29,30,31,32,33].

**Figure 1:**
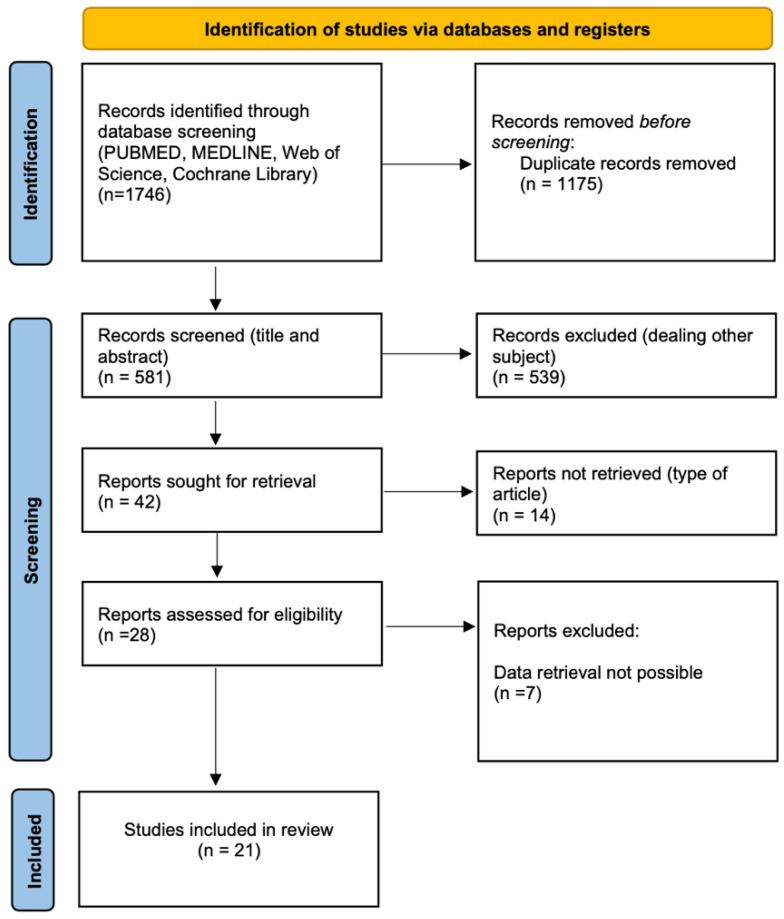
Systematic search process.

The systematic search process is summarised in Figure 1.

### 3.2. Study Characteristics and Quality Assessment

Articles were published between 2001 and 2022, including seven retrospective, three case series and eleven case reports with a total of 2873 patients. The average NOS score was 7.3.

Characteristics of the studies on PCLC included in the review were summarised in Table 1.

### 3.3. Pooled Analysis

#### 3.3.1. Baseline Characteristics

PCLC occurs mainly in males (57%) and at a median age between 52 and 66 (range: 24–82). Sixty-four percent of patients were former smokers. Among LC, the incidence of PC was 1.5%. Ascites was present in 63% of patients.

Regarding pTNM staging at diagnosis, 0.1% of patients were stage II, 0.3%% were stage III and the vast majority (99.6%) were stage IV. Peritoneum was the sole metastatic site in 13.5% of patients and it was synchronous in 94% of cases while metachronous in 6%. Concurrent metastatic sites were pleura in 29% of patients, liver in 20%, bone in 15%, adrenal glands and contralateral lung in 9%, distant nodes in 4%, pericardium in 2%, and small bowel, colon and eye in 0.5% of patients. Hystology was adenocarcinoma in 46% of patients, non-small-cell lung cancer (NSCLC) in 25% of patients, squamous cell carcinoma in 10% of patients and unspecified in 14%. EGFR mutation was present in 39% of patients, ALK and kRAS in 8%, MET in 3%, ROS in 2% and 40% had no mutations at all.

Baseline characteristics of patients with PC from LC are described in Table 2.

#### 3.3.2. PCLC Management and Outcomes

Adjuvant chemotherapy was indicated in 27% of patients in the form of cytotoxic agents in 56% of cases, EGFR/ALK-tyrosine kinase inhibitors in 40%, bevacizumab in 22%, platinum-based agents in 20% and immune-checkpoint inhibitors in 4%.

Recombinant human endostatin, BRAF-tyrosine kinase inhibitors, MEK-tyrosine kinase inhibitors and dendritic cell immunotherapy were indicated in 2% of patients. Surgery was indicated in 7% of cases. The median overall survival (OS) from lung cancer diagnosis was between 9 and 21 months (range: 1–88 months) while from the onset of PC, from 0.5 to 5 months (range 0–78) and 6% of patients were dead at latest follow-up.

PC from LC management and outcomes are summarised in Table 3.

### 3.4. Synchronous and Metachronous PCLC Characteristics Subanalysis

Synchronous and metachronous PCLC patient characteristics are described in Table 4. No comparative analysis was performed due to data paucity.

## 4. Discussion

The present study analysed the management and prognosis of patients with PCLC. PCLC appears to be a rare diagnosis; most patients do not receive any form of medical (chemotherapy) or surgical treatment and prognosis is generally very poor.

The pathogenesis of PCLC is not entirely clear, with Patil et al. finding a significant association with malignant pleural effusion, suggesting a possible route of spread, maybe through serosal communication [20]. In fact, in this review, as many as 29% of patients had concurrent pleural disease and this was the most prevalent concomitant site. Nonetheless, this association does not fully explain the pathogenesis, as the majority of PCLC patients never develop pleural disease.

The incidence of PCLC was 1.6% of LC patients and although low, it appears to be rising, as much as three times more than reported by older series, such as Satoh et al. and Flanagan et al. [13,25]. This increase in incidence may simply be the result of improved diagnostic modalities, or it may represent a new trend indicating that we will have to face this situation more often in the future. The latter idea may be supported by autopsy reports which find PCLC in 2% to 16% of cases [29]. In any case, the problem seems to deserve greater attention. This is particularly true when considering that LC is the most common adult cancer, and that the literature in this regard is scarce and provides generally low-quality evidence: this systematic review only found 21 articles on the matter, fourteen of which were case reports or small case series.

The average survival of patients with PCLC ranged between 0.5 and 5.2 months. This is in line with the paper by Niu et al. in which uncommon metastatic sites appear to have worse prognosis [2]. Nonetheless, attempts of PCLC treatment other than supportive management were rare: only 27% of patients received first-line or palliative chemotherapy and only 7% underwent surgery. This may be due to the poor performance status of these patients, but at first glance these numbers look very low and prompt the question of whether we are really doing all we can to help our patients. In particular, there appear to be situations in which the prognosis may be more favourable, and a more “aggressive” oncological management may pay off [25,34].

Patients with isolated PCLC had similar survival rates to patients with isolated “other organ” metastases in a study by Lurvink et al., despite (surprisingly!) significantly lower rates of systemic treatment. In the aforementioned study, these patients had 1- and 2-year OS rates of 22% and 10.5%, respectively [31].

Furthermore, LC may harbour mutations that may be antagonized by new-generation targeted therapy. Currently there are limited data on the specific genomic profile of PCLC. Authors reported EGFR and KRAS mutations, ALK rearrangements and rarely MET mutations. EGFR was the most commonly detected mutation in PCLC (40%) and there are several reports of response to specific tyrosine kinase inhibitors such as gefitinib, afatinib and erlotinib [20,35]. Furthermore, other studies show response to EGFR tyrosine kinase inhibitors without knowledge of their EGFR status [36,37]. Bevacizumab-based treatment may also be an effective treatment strategy for ascites management [29]. On the other hand, immune checkpoint inhibitors that yield good results in specific LC sub-populations seem to fare much worse in patients with PCLC [36,37].

Finally, a proper comparison between metachronous and synchronous PCLC was not possible, even if data may suggest synchronous PCLC to occur at an older age, without other metastases associated. Future studies should investigate this aspect to shed light on different characteristics and prognostic features regarding PCLC timing. Patients with metachronous metastases may have better chances of survival compared with those who had synchronous primary LC and PCLC diagnosis [29]. Unfortunately, most reported PCLC (94%) seem to be synchronous cases; although, this result may have been biased by the fact that the largest study (by far) included in this review focused exclusively on synchronous PCLC [31]. Other favourable prognostic factors seem to be younger age, female sex, and non-smoker status [29,31].

All these subcategories of PCLC may have longer survival times, and further studies should evaluate if they could gain major benefits from earlier diagnosis and adjunctive therapy. A quite good result was reported by Sibio et al., who treated two cases of PCLC with cytoreductive surgery, leading to survivals of 29 and 36 months after surgery, with the latter still being alive [27].

The approach utilised was directly derived from experience in PC from different abdominal (and non-abdominal) primary cancers. Cytoreductive surgery alone was found to be the main contributor to prolonged survival in colorectal cancer, proving the value of elimination gross malignant disease for improving prognosis [38,39,40]. Furthermore, association between cytoreductive surgery and HIPEC or, more recently, PIPAC are established surgical strategies for appendiceal carcinoma, colorectal and gynaecological (particularly ovarian) cancers, and may be considered in hyper-selected cases [4]. On this basis, there may be an argument for the investigation of a more active approach to the care of these patients, in an effort to offer them a chance of longer survival and possibly with a better quality of life. In fact, PCLC may lead to abdominal pain and partial or complete bowel obstruction, which may significantly worsen quality of life and may also deserve surgical treatment. Bowel obstruction is the most frequent clinical presentation in patients with gastrointestinal metastases [4]. Further studies should clarify the possible role of cytoreductive surgery especially when surgery for bowel obstruction seems unavoidable. Nonetheless, these aggressive treatments do have complications and the decision for such a commitment should be taken by a multidisciplinary team after careful consideration of the individual patient situation in terms of performance status and tumour biology (mutations) and distribution (other metastatic sites, peritoneal cancer index—PCI, etc.) [41,42].

Early diagnosis could allow treatment of a less extensive disease (lower PCI) and therefore also be important. In the setting of a growing PCLC incidence, it will be important to be keep a high index of suspicion.

This study has some limitations, the main one being the scarcity and quality of the literature, in addition, another one is the presence of one study reporting on the majority of patients included and reporting on only synchronous PCLC.

Overall, PCLC appears to be a rising diagnosis, with a poor prognosis and limited therapeutical interventions. A more aggressive approach may obtain improved results in some patients and should probably be investigated further in the near future. As the incidence is low, appropriate national or international registries should be encouraged.

## 5. Conclusions

The literature on PCLC is scarce. Its incidence is low but appears to be substantially rising and is likely to be an underestimation. Prognosis is very poor and therapeutic strategies have been limited and used in a minority of patients. Subcategories of PCLC patients may have an improved prognosis and may benefit from an aggressive oncological approach, including cytoreductive surgery. Further investigation would be needed in this regard.

## Figures and Tables

**Table 1:** Characteristics of studies included in the review.

Authors	Year	Country/Region	Journal	Study Design	N° Patients	NOS Score
Satoh et al. [13]	2001	Japan	Oncology Reports	Retrospective	12	7
Kimura et al. [14]	2008	Japan	Journal of Medical Case Reports	Case Report	1	-
Su et al. [15]	2008	Taiwan	Respirology	Retrospective	30	7
Tanriverdi et al. [16]	2012	Turkey	Wspolczesna Onkol	Case Report	1	-
Sereno et al. [17]	2013	Spain	Oncology letters	Case Series	4	-
Bazine et al. [18]	2014	Morocco	Case Reports in Oncology	Case Report	1	-
Li et al. [19]	2014	China	Oncology letters	Case Report	1	-
Patil et al. [20]	2016	Colorado	Lung Cancer	Retrospective	33	7
Kobayashi et al. [21]	2016	Japan	Respirology Case Reports	Case Report	1	-
Hanane et al. [22]	2016	Morocco	PanAfrican Medical Journal	Case Report	1	-
Yang et al. [23]	2017	China	Journal of Medical Case Report	Case Report	1	-
Kamaleshwaran et al. [24]	2017	India	Indian Journal of Nuclear Medicine	Case Report	1	-
Flanagan et al. [25]	2018	Ireland	European Journal of surgical oncology	Retrospective	139	8
Jui-Feng Hsu et al. [26]	2018	Taiwan	Asia-Pacific Journal of Clinical Oncology	Case Series	3	-
Sibio et al. [27]	2019	Italy	Journal of Medical Case Reports	Case Series	2	-
Kawaguchi et al. [28]	2019	Japan	Clinical Case Reports	Case Report	1	-
Abbate et al. [29]	2019	Italy	Future Oncology	Retrospective	60	7
Kazakova et al. [30]	2020	USA	Unusual presentation of more common disease/injury	Case Report	1	-
Lurvink et al. [31]	2021	Netherlands	Clinical & Experimental Metastasis	Retrospective	2533	8
Tani et al. [32]	2021	Japan	Cancer Management and Research	Retrospective	46	7
Yagami et al. [33]	2022	Japan	Oncotargets and therapy	Case report	1	-

**Table 2:** Baseline characteristics of peritoneal carcinosis from lung cancer patients.

Authors	Number of Patients	Male (*n* %)	Age Median (Range) (Years)	Smoker (*n* %)	Incidence of PC (*n* %)	Time from Diagnosis to PC Median (Range) (Months)	Ascites (*n*%)	Stage at Diagnosis (*n* %)	Other M (*n* %)	Peritoneal Single M Site (*n*, %)	Histological Type (*n* %)	Mutations (*n*, %)	Clinical Presentation (*n* %)
Satoh et al. [13]	12	6 50%	54 (34–74)	na	12/1041 1.2%	9 (0–36)	na	na	Pleura 9 75% Lung 6 50% Liver 4 33.3% Bone 5 41.7% Brain 3 25% Distant node 3 25%	na	Adenocarcinoma 7 58.3% SCLC 1 8.3% SCC 2 16.7% NSCLC 2 16.7%	na	Synchronous 1 8.3% Metachronous 11 91.7%
Kimura et al. [14]	1	0 0%	52	na	na	25	1 100%	IV	Lung 1 100% Pleura 1 100%	na	Adenocarcinoma 1 100%	na	Metachronous 1 100%
Su et al. [15]	30	20 66.7%	59 (29–83)	na	na	8.5 (0–38)	30 100%	IIIb 6 20% IV 24 80%	Lung 9 30% Liver 11 36.7% Bone 13 43.3% Brain 5 16.7% Pleura 24 80% Adrenal 3 10% Soft tissue 1 3.3% Eye 1 3.3% Pancreas 2 6.7% Pericardial effusion 3 10% Spleen 1 3.3%	0%	Adenocarcinoma 25 83.4% SCLC 3 10% SCC 1 3.3% Mixed small cell/squamous cell carcinoma 1 3.3%	na	na
Tanriverdi et al. [16]	1	1 100%	59	1 100%	na	3	1 100%	IIIa 1 100%	Pericardium 1 100%	0%	Adenocarcinoma 1 100%	na	Metachronous 1 100%
Sereno et al. [17]	4	3 75 %	64 (52–67) Mean 61.5	2 100%	na	3 (0–12)	1 25%	IVb 1 25% (1 pt)	Lung 1 25% Liver 1 25% Pleura 3 75% Adrenal gland 1 25%	0%	Adenocarcinoma 4 100%	EGFR 2 50%	Metachronous 3 75% Synchronous 1 25%
Bazine et al. [18]	1	0 0%	55	0 0%	na	0	na	na	0 0%	1 100%	Adenocarcinoma 1 100%	None 1 100%	Synchronous 1 100%
Li et al. [19]	1	1 100%	63	1 100%	na	0	1 100%	na	0 0%	1 100%	SCC 1 100%	BRAF 1 100% kRAS 1 100%	Synchronous 1 100%
Patil et al. [20]	33	12 36%	58 (51–91)	13 39%	33/410 8%	16.5 (0.6–108)	na	na	Lung 5 15% Liver 3 9% Bone 14 42% Brain 10 30% Pleura 26 79% Adrenal 4 12% Soft tissue 4 12%	na	NSCLC 33 100%	EGFR 17 51% kRAS 5 15% MET 1 3% ALK 5 15% None 5 15%	Metachronous 33 100%
Kobayashi et al. [21]	1	0 0%	61	0 0%	na	na	1 100%	IV 100%	Lung 1 100% Pleura 1 100%	0 0%	Adenocarcinoma 1 100%	EGFR 1 100%	Metachronous 1 100%
Hanane et al. [22]	1	1 100%	56	1 100%	na	14	1 100%	IIIa 1 100%	0 0%	1 100%	Adenocarcinoma 1 100%	None 1 0%	Metachronous 1 100%
Yang et al. [23]	1	1 100%	82	1 100%	na	1.7	1 100%	IIIa 1 100%	0 0%	1 100%	SCC 1 100%	kRAS 1 100%	Metachronous 1 100%
Kamaleshwaran et al. [24]	1	1 100%	45	na	na	0	na	IV 1 100%	0 0%	1 100%	NSCLC 1 100%	EGFR 1 100%	Synchronous 1 100%
Flanagan et al. [25]	139	80 57%	na	na	139/41,789 0.3%	8.5 (1–9)	na	IV 139 100%	Liver 37 26.6% Bone 10 7.2% Brain 9 6.5% Distant node 8 5.8% Adrenal 18 12.9%	34 24.4%	Adenocarcinoma 51 37% SCLC 27 19% SCC 21 15% NSCLC 12 9% Unspecified 28 20%	na	Synchronous 99 71 % Metachronous 40 29 %
Jui-Feng Hsu et al. [26]	3	2 67%	66 (53–67) Mean 62	1 33.3%	3/265 1.1%	21 (0–28) Mean 16.3	3 100%	IV 3 100%	Liver 1 33.3% Pericardium 1 33.3%	2 66.7%	Adenocarcinoma 3 100%	EGFR 3 100%	Synchronous 1 33.3% Metachronous 2 66.7%
Sibio et al. [27]	2	2 100%	52 (44–59)	1 50%	na	42 (36–48)	1 50%	IIb 2 100%	Brain 1 50% Colon 1 50% Small bowel 1 50% Spleen 1 50%	0 0%	Adenocarcinoma 2 100%	na	Metachronous 2 100%
Kawaguchi et al. [28]	1	1 100%	42	1 100%	na	21	1 100%	IV 1 100%	Lung 1 100% Brain 1 100%	0 0%	Adenocarcinoma 1 100%	EGFR 1 100%	Metachronous 1 100%
Abbate et al. [29]	60	na	60 (25–75)	43 72%	na	na	na	na	na	na	Adenocarcinoma 48 80% SCC 1 2% Unspecified 11 18%	EGFR 7/23 30% ALK 3/17 18% MET 2/4 50% ROS 1/3 33% 3	Synchronous 20 33.3% Metachronous 40 66.7%
Kazakova et al. [30]	1	1 100%	56	0 0%	na	0	1 100%	IV 1 100%	0 0%	1 100%	Adenocarcinoma 1 100%	ROS1 1 100%	Synchronous 1 100%
Lurvink et al. [31]	2533	1483 58.5%	Mean 67 ± 10	na	2533/129,651 2%	0	na	IV 2533 100%	na	326 12.9%	Adenocarcinoma 1122 44.3% SCLC 500 19.7% SCC 258 10.2% NSCLC 653 25.8%	na	Synchronous 2533 100%
Tani et al. [32]	46	33 71.7%	66 (59–71)	36 78%	na	na	15 32.6%	na	Brain 5 10% Pleural 17 37%	na	Adenocarcinoma 40 87% NSCLC 4 8.6% SCC 1 2.2% Pleomorphic carcinoma 1 2.2%	EGFR 14 30.4% ALK 1 2.2% None 31 67.4%	Synchronous 12 26.1% Metachronous 34 73.9%
Yagami et al. [33]	1	1 100%	67	0 0%	na	33	1 100%	I 100%	Pleural 1 100%	0 0%	Adenocarcinoma	BRAF 100%	Metachronous 1 100%
Total	2873	1649/2873 57.4%	Median range 52–66 (range 25–91)	101/157 64.3%	2720/173,476 1.5%	Median range 0–42 m (range 0–108)	59/94 63%	II 2/2873 0.1% III 9/2873 0.3% IV 2706/2720 99.6%	Pleural 81/280 28.9% Liver 57/280 20.4% Bone 42/280 15% Brain 34/280 12.1% Adrenal 26/280 9.3% Lung 24/280 8.6% Distant node 11/280 3.9% Pericardium 5/280 1.9% Soft tissue 5/280 1.9% Pancreas 2/280 0.7% Spleen 2/280 0.7% Small bowel 1/280 0.4% Colon 1/280 0.4% Eye 1/280 0.4%	368/2721 13.5%	Adenocarcinoma 1310/2873 45.6% NSCLC 705/2873 24.5% SCLC 531/2873 18.5% SCC 286/2873 10% Pleomorphic carcinoma 1/2873 0.03% Mixed small cell/squamous cell carcinoma 1/2873 0.03% Unspecified 39/2873 13.6	EGFR 46/117 39.3% ALK 9/111 8.1% kRAS 7/95 7.5% MET 3/98 3.1% ROS 2/97 2.1% BRAF 2/95 2.1% None 38/95 40%	Synchronous 2671/2843 94% Metachronous 172/2843 6%

PC: peritoneal carcinosis; M: metastases; SCLC: small-cell lung cancer; SCC: squamous cell carcinoma; NSCLC: non-small-cell lung cancer.

**Table 3:** Management and outcomes of peritoneal carcinosis from lung cancer.

Authors	Number of Patients	Chemotherapy (*n* %)	Type of Chemotherapy (*n* %)	Surgical Intervention (*n* %)	OS from Lung Cancer Diagnosis Median (Range) (Months)	OS PC Median/Mean (Range) (Months)	Death (*n* %)
Satoh et al. [13]	12	1 8.3%	Platin-based agents 1 8.3%	0 0%	na	2 (1–9)	12 100%
Kimura et al. [14]	1	1 100%	Dendritic cell immunotherapy 1 100%	0 0%	35	10	0%
Su et al. [15]	30	9/25 36%	na	0 0%	9 (0.2–42.7) (25 pt)	0.5 (0–11.3) (25 pt)	25/26 96.1%
Tanriverdi et al. [16]	1	1 100%	Docetaxel 1 100%	0 0%	na	2	1 100%
Sereno et al. [17]	4	4 100%	Docetaxel 3 75% Pemetrexed 3 75% Carboplatin 3 75% Cisplatin 1 25% Paclitaxel 2 50% Bevacizumab 2 50% Erlotinib 2 50%	0 0%	na	na	1 25%
Bazine et al. [18]	1	1 100%	Pemetrexed 1 100% Carboplatin 1 100% Paclitaxel 1 100% Bevacizumab 1 100%	0 0%	10	10	0 0%
Li et al. [19]	1	0 0%	na	0 0%	0.2	0.2	1 100%
Patil et al. [20]	33	na	na	0 0%	20.5 (1–88)	2 (0–78)	33 100%
Kobayashi et al. [21]	1	1 100%	Afatinib 1 100%	0 0%	na	12	0 0%
Hanane et al. [22]	1	1 100%	Gemcitabine 1 100% Cisplatin 1 100% Bevacizumab 1 100%	0 0%	na	6	1 100%
Yang et al. [23]	1	1 100%	Cisplatin 1 100% Recombinant human endostatin 1 100%	0 0%	2.1	0.4	1 100%
Kamaleshwaran et al. [24]	1	1 100%	Erlotinib 1 100%	0 0%	na	na	na
Flanagan et al. [25]	139	50 35%	na	11 7%	10	1.3 (0–16.2)	139 100%
Jui-Feng Hsu et al. [26]	3	3 100%	Gemcitabine 1 33.3% Bevacizumab 3 100% Erlotinib 1 33.3% Afatinib 1 33.3%	0 0%	**65.6**	**41.3**	1 33.3%
Sibio et al. [27]	2	2 100%	Cisplatin 1 50% Gemcitabile 1 50%	2 100%	74.5 (65–84)	**32.5 (29–36)**	1 50%
Kawaguchi et al. [28]	1	1 100%	Osimertinib 1 100%	0 0%	25	4	1 100%
Abbate et al. [29]	60	58 96.7%	na	0 0%	17.5	3.5	na
Kazakova et al. [30]	1	1 100%	Crizotinib 1 100%	0 0%	6	6	0 0%
Lurvink et al. [31]	2533	590 23.3%	na	189 7%	na	2.5	na
Tani et al. [32]	46	25 54.3%	Cytotoxic agents 13 28.3% EGFR/ALK-tyrosine kinase inhibitors 10 21.7% Immune-checkpoint inhibitors 2 4.3% Bevacizumab 3 6.5%	0 0%	na	5.2 (2.1–6.3)	na
Yagami et al. [33]	1	1 100%	Dabrafenib 1 100% Trametinib1 100%	0%	33	40	0 %
Total	2873	753/2835 26.6%	Cytotoxic agents 25/45 55.5% EGFR/ALK-tyrosine kinase inhibitors 18/45 40% Bevacizumab 10/45 22.2% Platinum based agents 9/45 20% Immune-checkpoint inhibitors 2/45 4.4% Recombinant human endostatin 1/45 2.2% BRAF-tyrosine kinase inhibitors 1/45 2.2% MEK-tyrosine kinase inhibitors 1/45 2.2% Dendritic cell immunotherapy 1/45 2.2%	202/2873 7%	Median range 9–20.5 (range 0.1–88)	Median range 0.5–5.2 (range 0–78)	220/229 96.1%

OS: overall survival. Bold font represents mean values.

**Table 4:** Synchronous and metachronous PCLC patient characteristics.

	Male (*n* %)	Age Mean (Years)	Smoker (*n* %)	Ascites (*n*%)	Stage at Diagnosis (*n* %)	Other M (*n* %)	Peritoneal Single M Site (*n*, %)	Histological Type (*n* %)	Mutations (*n*, %)
Synchronous (*n* = 2671)	1488/2538 59%	67	1/5 20%	3/4 75%	IV 2671/2671 100%	Pleura 1/6 17%	5/6 83%	Adenocarcinoma 1126/2539 44% NSCLC 654/2539 26% SCLC 500/2539 20% SCC 259/2539 10%	None 2/6 33% EGFR 2/6 33% BRAF 1/6 17% kRAS 1/6 17% ROS1 1/6 17%
Metachronous (*n* = 172)	22/47 47%	58	21/46 46%	12/14 86%	II 2 1.2% III 9 5.2% IV 161 93.6%	Pleura 31/47 66% Bone 14/47 30% Brain 12/47 26% Lung 9/47 19% Adrenal gland 5/47 11% Soft tissue 4/47 8% Pericardium 2/47 4% Bowel 2/47 4%	3/46 7%	NSCLC 33/47 70% Adenocarcinoma 13/47 28% SCC 1/47 2%	None 6/43 14% EGFR 23/43 53% kRAS 6/43 14% ALK 5/43 12% BRAF 1/43 2% MET 1/43 2%

## Data Availability

All data generated or analysed during this study are included in this published article. The dataset supporting reported results is protected and access availability must be obtained.
